# The role of government fiscal and tax incentives in green technology innovation and enterprise development: implications for human health and hygiene

**DOI:** 10.3389/fpubh.2024.1502856

**Published:** 2025-01-08

**Authors:** Yinghong Shi, Jing Ge

**Affiliations:** College of Business Administration, Chongqing Vocational and Technical University of Mechatronics, Chongqing, China

**Keywords:** waste treatment, public health, green technology, environmental protection, fiscal and tax incentives

## Abstract

**Purpose:**

This study aims to examine how government fiscal and tax incentives facilitate the development and application of green technologies, promoting corporate environmental responsibility and improving public health and hygiene.

**Methods:**

The study utilizes empirical data from listed enterprises in the new energy automobile industry between 2018 and 2023. A multiple regression model is used to assess the effects of government subsidies and tax incentives on green technological innovation and enterprise growth, controlling for various factors such as enterprise size and R&D investment.

**Results:**

The analysis reveals that both government subsidies and tax incentives have a significant positive impact on green technology innovation and the development of green enterprises. The results also highlight regional variations, with enterprises in central and western regions benefiting more from tax incentives than those in eastern regions.

**Conclusion:**

Government fiscal and tax incentives play a crucial role in fostering green technology innovation and sustainable enterprise development. To optimize the effectiveness of these policies, improvements in policy implementation, especially in addressing regional disparities, are necessary. This study provides insights for policymakers and green enterprises on leveraging incentives for long-term sustainability and public health benefits.

## 1 Introduction

With the continuous development of industrialization, science, and technology, an increasing number of industrial products have become integral to people’s lives. However, this progress has been accompanied by a growing volume of industrial waste, particularly new types of industrial waste (1). Without timely and safe treatment or recycling, industrial waste can significantly harm the environment, pose serious risks to public health, and threaten human health and ecological safety (2). In response, many countries are now pursuing a low-carbon and green economy. Enterprises in these nations are adopting green environmental protection technologies, with some focusing on the development and innovation of such technologies. Green technology innovation encompasses both “environmental protection” and “innovation.” It offers multiple benefits: reducing environmental pollution and lowering costs associated with governmental environmental regulations (3), improving production efficiency, promoting industrial optimization and upgrading, enhancing competitive advantages, and supporting the long-term, stable development of enterprises. However, similar to traditional innovation, green technology innovation also has certain externalities (4). For instance, capital investment in green product production and process innovation will inevitably divert resources from other areas (5).

Meanwhile, the research and development (R&D) cycle is long, and the return is time-sensitive, which will affect the circulation of capital of the enterprise (6). On the other hand, innovation has spillover (7), and the R&D achievements of enterprises are easy to obtain by other enterprises at a lower cost, which threatens their competitive position and is not conducive to the production and development of enterprises (8). Therefore, due to the complexity, the advanced development process of green environmental protection technology has characteristics such as a long cycle, high risk, uncertainty factors, etc. Moreover, green environmental protection technology serves more public than private welfare (9). Therefore, the risks that enterprises bear are quite high in the exploitation of green environmental protection technology and the corresponding research and development activities, for which it is difficult to obtain financial institutions’ financing support (10). Consequently, compared with green technology, enterprises are more inclined to use traditional technology with high energy consumption, high pollution, and low cost.

Government departments will generally issue fiscal and tax incentive policies to encourage green enterprises to conduct research and development and apply green environmental protection technology to encourage enterprises to innovate in production, technology, personnel, and other aspects (11, 12). The government issues fiscal and tax incentive policies, such as tax incentives, financial subsidies, and R&D awards, which can improve enterprise participation in innovation and R&D activities related to green and environmental technologies to a certain extent. These policies encourage enterprises to actively undertake and fulfill environmental responsibilities and improve performance (13). However, limited by factors such as the hysteresis effect of market development and management efficiency of the government department, government departments of fiscal and tax incentives cannot directly and efficiently solve the problem of enterprise funds (14). As a result, green enterprises need to actively seek government policy support in the innovation research and development process of green technology and the promotion of green products to save their own R&D funds to the greatest extent and enhance their corporate image and market core competitiveness.

This study analyzes the basic content and form of the government tax incentive system, summarizes the government tax incentives for enterprises to develop a positive impact, and analyzes the difference between green innovation and tradition. Thus, it is concluded that government fiscal and tax incentives are necessary for green enterprises to operate innovation research of green technology and enterprise development. It also analyzes the possible problems in the implementation of the government’s fiscal and taxation system. Based on this, it analyzes the mechanisms and strategies that green enterprises should adopt in the innovation and development of green environmental protection technology and enterprise development.

The innovative aspects of the study include the following:

1. Comprehensive analysis of government incentives: The study examines fiscal subsidies and tax incentives, offering a holistic perspective on how these policies influence green technology innovation and enterprise growth.
2. Use of empirical data: By utilizing empirical data from listed enterprises in the new energy automobile industry between 2018 and 2023, the study provides analysis based on real-world market data, enhancing the practical application of its findings.
3. Application of multiple regression models: The study employs multiple regression models to control for various factors such as enterprise size and R&D investment, allowing for a more accurate assessment of the impact of government incentives.
4. Exploration of regional variations: The study reveals the benefits enterprises in different regions (eastern, central, and western) derive from tax incentives, offering nuanced insights for policymakers.

## 2 Government fiscal and tax incentives under a low-carbon economy

### 2.1 Overview of government fiscal and tax incentives

Government fiscal and tax incentives primarily constitute the incentive mechanisms for economic activities and behaviors, such as consumption, production, and investment, which are generated by the government through tax incentives, focusing on production activities (15). The scope and application fields of these tax incentives are extensive and can be broadly categorized into general domestic production incentives (16), special domestic production incentives (17), foreign trade incentives (18), and others. General domestic production incentives encompass measures like tax holidays (providing a certain period of tax exemption at the start of business operations), encouragement of profit retention (offering tax relief on undistributed profits), and accelerated depreciation to stimulate investment. Special domestic production incentives are tailored to provide specific encouragement and stimulation to key sectors and emerging industries that are vital to the national economy and regions requiring differentiated treatment for their development. Foreign trade incentives are designed to bolster the export of goods and encourage foreign investment. For example, key sectors and emerging industries in the national economy are given special tax reduction and exemption treatment, and necessary regions are treated differently and given special encouragement. Foreign trade incentives encourage the export of goods and foreign investment.

Tariff exemptions play a crucial role in reducing the costs associated with importing advanced technologies and equipment that green enterprises need. For instance, when enterprises are exempted from tariffs on imported solar panels or wind turbines, they can significantly lower their capital expenditures. This enables them to allocate more resources toward research and development (R&D) initiatives, facilitating the adoption of innovative green technologies. Accelerated depreciation policies benefit green technology enterprises by allowing them to quickly recover their investments. For example, when an enterprise invests in energy-efficient machinery, the ability to depreciate these assets at an accelerated rate reduces taxable income in the initial years. This not only improves cash flow but also incentivizes further investments in sustainable technologies.

**Figure 1:**
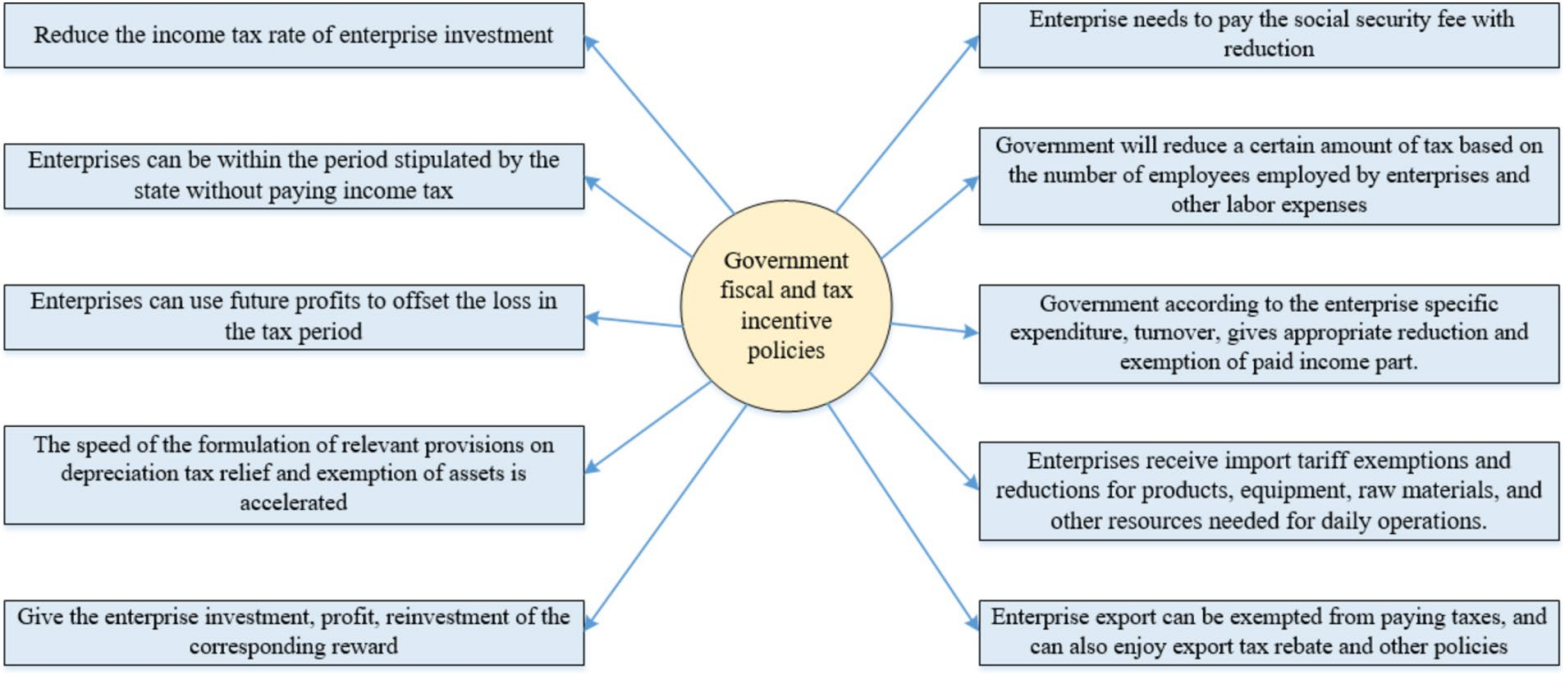
Contents of government fiscal and tax incentive policies.

The government’s tax incentive policies for enterprise innovation mainly include tax cuts (19), fiscal incentives (16), and other related measures (20), recognizing that enterprises need to invest significant funds for daily management and operations. The government rewards investment enterprises by exempting income tax for a specific period. Tax relief is granted based on factors such as the proportion of exports, product output, and employment. The government offers additional incentives if an enterprise’s contribution is exceptional (21). The methods used to implement fiscal and tax incentive policies are shown in Figure 1 and include the following: First is to reduce the income tax rate of enterprise investment (22). Second, enterprises can be within the period stipulated by the state without paying income tax (23). Third, the enterprise can use future profits to offset the loss in the tax period (24). Fourth, the speed of the formulation of relevant provisions on depreciation tax relief and exemption of assets is accelerated (25). The fifth is to give the enterprise investment, profit, and reinvestment of the corresponding reward (26). Sixth, the enterprise needs to pay the social security fee with a reduction (27). Seventh, the government will reduce a certain amount of tax based on the number of employees employed by enterprises and other labor expenses (28). Eighth, according to the enterprise-specific expenditure and turnover, the government gives appropriate reduction and exemption of paid income part. Ninth, enterprises receive import tariff exemptions and reductions for products, equipment, raw materials, and other resources needed for daily operations. Finally, enterprise exports can be exempted from paying taxes and can also enjoy export tax rebates and other policies (29).

### 2.2 Positive impact of government fiscal and tax incentives in low-carbon economy

**Figure 2:**
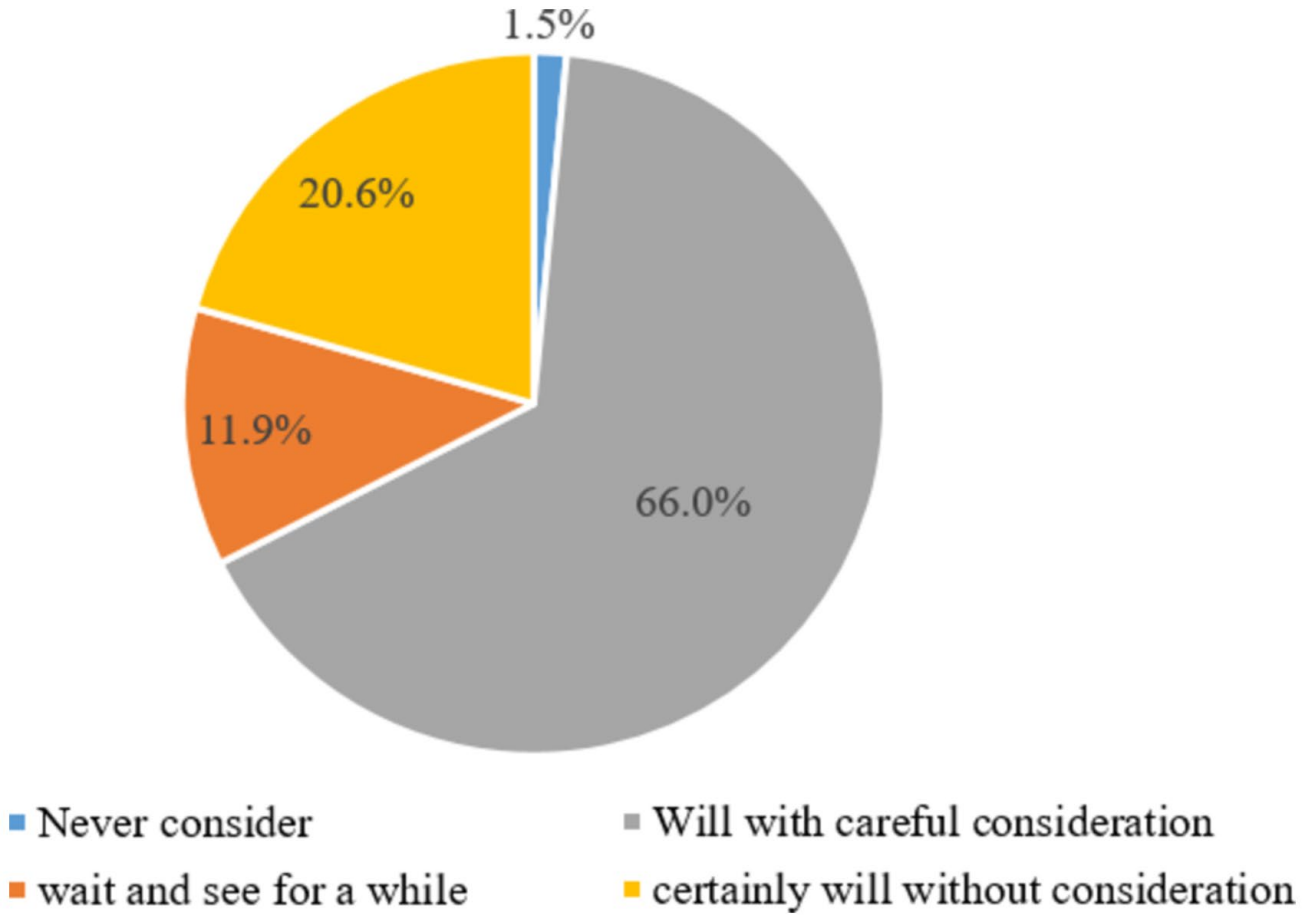
A survey on the willingness of enterprises to transform into green production mode.

A low-carbon economy is a win-win model for economic and social development and ecological protection (30). It aims to reduce high carbon energy consumption from coal and oil while also decreasing greenhouse gas emissions. This approach is guided by the principles of sustainable development and is achieved through technical innovation, system innovation, industrial transformation, the development of new energy sources, and other means. The low-carbon economic development model has a long benefit period and a large initial investment, which puts a certain burden on the innovation and development of enterprises. The government can effectively help enterprises carry out green environmental protection technology innovation and enterprise development through fiscal and tax incentives (31). As shown in Figure 2, more than 86.6% of enterprises are willing to transform into green production mode if government fiscal and tax incentive policies support their innovation and development.

First, government fiscal and tax incentive policies can reduce production costs and improve production efficiency. Modern enterprises, especially green enterprises, need to introduce advanced technology and equipment for their development. In this process, the government will give part of its fiscal revenue to enterprises through fiscal and tax incentives so that enterprises can increase their funds for innovation and development and enhance their core competitiveness. When addressing new types of waste, enterprises need to develop or purchase the corresponding technology and equipment. The government can offer additional deductions for R&D and accelerated depreciation on research and development equipment, thus reducing the costs associated with innovation (32). Additionally, imported technologies and equipment may be exempt from tariffs. Thus, the fiscal expenditure of enterprises can be reduced, and R&D funds can be increased.

Second, government fiscal and tax incentive policies can reduce the risk of enterprise operation. The government can deduct the losses incurred by enterprises after the occurrence of risks before tax, transfer part of the risks borne by enterprises to itself, and greatly reduce the tax burden of enterprises (33). In this way, the risks that enterprises may face in innovation and development will be reduced, thus encouraging enterprises to innovate and create. For example, consider the treatment of newly emerging waste. When the newly emerging waste needs to be treated with environmental protection, the enterprise may face the risk of failure in the research and development of technology, which is a huge consumption of the operating cost of the enterprise. After repeated failures, the enterprise is likely to give up research. The government can provide support for the innovation and development of enterprises by giving corresponding incentives to enterprises in terms of investment, profit, and reinvestment to ensure that enterprises’ risks in carrying out innovation activities are reduced and encourage enterprises to actively participate in innovation activities.

Third, government fiscal and tax incentive policies can promote enterprises to increase human capital investment. These policies also influence human resource management by allowing deductions for employee training expenses, wages, and salaries before tax, thereby affecting labor supply and demand within enterprises (34). Green enterprises need a large number of technical talents to carry out green environmental protection technology innovation and enterprise development, which requires more opportunities for employees to participate in training to improve the innovation ability and level of the enterprise. Regarding technical training, however, the cost is higher, and the enterprise staff liquidity is bigger, so the enterprise needs to consume large amounts of money on the training. If the enterprise sets its sights on short-term gains and does not pay attention to the cultivation of talent, it will be hard to retain and attract talent, and the innovation ability of enterprises will have a negative impact. Therefore, the government can reduce the labor cost of enterprises by giving certain pre-tax deductions for the training of employees of enterprises and providing certain tax subsidies for the introduction of technical talents. With the support of talents, the innovation level and ability of enterprises can be continuously improved, which is more conducive to the innovation and development of enterprises.

## 3 Empirical analysis of the impact of government fiscal and tax incentive policies on green technology innovation and green enterprise development

### 3.1 Research hypothesis

The government subsidies and tax incentives introduced by the state are important for the technological innovation activities of green enterprises, providing financial support for technological innovation. This reduces enterprises’ innovation costs and risks to a certain extent, stimulates their enthusiasm for innovation, and promotes innovation and development. Financial subsidies can stimulate enterprises to carry out R&D green technology innovation in the maturity period. Therefore, this study proposes the following hypotheses:

H1: A significant positive correlation exists between government subsidy policy, green technology innovation, and green enterprise development.

Tax incentives mainly act on the innovation costs and profits of enterprises. This is equivalent to the government giving up part of its tax revenue to reduce the level of tax burden on enterprises and increase their own funds, thus incentivizing them to carry out innovative activities. Therefore, the following hypothesis is proposed:

H2: There is a significant positive relationship between tax incentives, green technology innovation, and green enterprise development.

Government subsidy and tax preference policies are the means of macro-control of the market when the government faces market failure. These two types of policies are different in the way they work on enterprise innovation and research and development. Government subsidies play a role in the early stage of R&D innovation, which directly increases the cash flow of enterprises and makes up for the financial needs of early innovation, as well as R&D. Tax incentives are ex-post incentives. Tax incentives are ex-post incentives. The government transfers part of the benefits to the enterprise, increasing the enterprise’s motivation to carry out innovative R&D. Generally, both government subsidies and tax incentives can improve the enterprise’s green technology innovation development. Therefore, the following hypothesis is proposed:

H3: There is a significant positive correlation between government subsidies, tax incentives, green technology innovation, and green enterprise development.

The role of environmental regulations in fostering green technology innovation, as highlighted by Lv et al. (35) and Guo et al. (36), underscores the importance of considering the broader policy environment that influences the effectiveness of government subsidies and tax incentives. Their study suggests that stringent environmental regulations can catalyze green technology innovation, which aligns with our hypothesis that government policies, including fiscal and tax incentives, significantly influence green technology innovation and enterprise development.

### 3.2 Research design

#### 3.2.1 Variable selection

Measured by the number of invention patent authorizations, it reflects the quality and technological competitiveness of enterprises in technological innovation. Therefore, this study selects the number of invention patents of green technology as the explanatory variable. The explanatory variables are fiscal and tax incentive policies, including the two indicators of government subsidy policies and tax incentives. The control variables include enterprise size, R&D investment, current ratio, gearing ratio, economic environment, etc., as shown in Table 1.

**Table 1:** Variable definition table.

Variable type	Variable name	Variable symbol	Variable definition
Dependent variable	Innovation quality	patent	Number of granted patents
Independent variable	Government subsidy policy	sub	The ratio of government subsidy received by the enterprise
	Tax incentive policy	tax	Ratio of tax rebate received by the enterprise
	Enterprise size	size	Ratio of total assets
	R&D investment	rd	R&D expenditure of the enterprise
Control variable	Liquidity ratio	Cura	Liquid assets/liquid liabilities
	Asset-liability ratio	lev	Total liabilities/Total assets
	Economic environment	GDP	GDP of the region where the enterprise is located

#### 3.2.2 Model construction

To test the impact of government subsidies on green technology innovation and green enterprise development, this study verifies H1 by constructing a multiple regression model (Equation 1).


(1) $$\mathrm{inno}\_vqua_{xn}=\alpha +\gamma_{1}\cdot sub+\theta_{y}\cdot I_{xn}+\in_{xn}$$

Similarly, to test the impact of tax incentives on green technology innovation and green enterprise development, this study verifies H2 by constructing a multiple regression model (Equation 2).


(2) $$\mathrm{inno}\_vqua_{xn}=\alpha +\gamma_{1}\cdot \mathrm{tax}+\theta_{y}\cdot I_{xn}+\in_{xn}$$

A multiple regression model (Equation 3) is constructed to verify H3 to study the incentive effect of government subsidies and tax incentives on green technology innovation and green enterprise development.


(3) $$\mathrm{inno}\_vqua_{xn}=\alpha +\gamma_{1}\cdot sub+\gamma_{2}\cdot \mathrm{tax}+\theta_{y}\cdot I_{xn}+\in_{xn}$$

In the model, $\mathrm{inn}o_{vqua_{xn}}$ denotes the level of green technology innovation of enterprise *x* in the *n*-th year; *sub* denotes the government subsidy policy implemented by the government to enhance the technological innovation of green enterprises; *tax* denotes the tax incentives implemented; *x* denotes the *x*-th enterprise; *n* denotes the year; $I_{xn}$ denotes the control variables; $\theta_{y}$ denotes the coefficients of the control variables; $\gamma_{1}$ and $\gamma_{2}$ represents the regression coefficients of the government subsidy (sub) and the tax incentives (tax), respectively; α denotes the intercept term; and $\varepsilon_{xn}$ denotes the random perturbation term.

### 3.3 Sample selection and data source

To ensure the availability of data, this study selects the new energy automobile industry as the representative industry of the “green technology innovation industry.” Therefore, this study selects listed enterprises in the new energy automobile industry as samples, and the study period is 2018–2023. The number of authorized patents for technological innovation is from the China Research Data Service Platform (CNRDS), and the rest of the data are from the Wind database. To ensure the authenticity of the data, the sample data with missing variables were excluded from this study. The 2,188 observations whose conditions were met were screened according to the criteria, and then the continuous-type variables were subjected to 1% shrinkage. The data were processed and analyzed mainly using Stata15.

## 4 Empirical tests and analysis

### 4.1 Descriptive statistical analysis

In this study, descriptive statistical analysis was carried out on the relevant variables of 2,188 sample enterprises from 2018 to 2023, and the results were obtained as shown in Table 2.

**Table 2:** Descriptive statistics for each variable.

Variables	Mean	Sd	Min	Max
patent	28.723	129.316	0	2,395
sub	11.325	8.634	0	20.559
tax	16.214	6.554	0	22.534
size	23.437	2.318	20.911	26.871
rd	19.156	3.987	0	23.688
cura	3.135	2.514	0.611	10.865
lev	0.557	0.199	0.193	0.995
GDP	30.401	0.751	28.431	31.261

Table 2 shows that the mean value of high-quality innovation output of green low-carbon enterprises is 28.723, but the standard deviation is 129.316. The gap between the minimum and maximum values is very large, indicating that there are large differences in the innovation and technology level of listed enterprises in the new energy automobile industry and that the ability of high-quality innovation output is uneven. The mean value of government subsidies (sub) is 11.325, and the standard deviation is 8.634, indicating that government subsidies are “universal” for the green and low-carbon industry, and there is little difference in the amount of government subsidies received by enterprises. The mean value of tax incentives is 16.214, and the standard deviation is 6.554. Compared with government subsidies, the difference in the amount of tax incentives enterprises receive is relatively small. In terms of control variables, the mean value of enterprise size is 23.437, and the standard deviation is 2.318, with small fluctuation, indicating that the difference in the size of sample enterprises is relatively small. The mean value of research and development investment (RD) is 19.156, and the standard deviation is 3.987, indicating that the sample enterprises do not differ much in the amount of research and development investment. The mean value of the current ratio is 3.135, and the standard deviation is 2.514, indicating that the difference in the solvency of the sample firms is not too obvious. The mean value of the gearing ratio is 0.557, and the standard deviation is 0.199, indicating that the gearing ratio of the sample enterprises is within a reasonable range, and there is not much difference between different sample enterprises.

### 4.2 Correlation analysis

Because the covariance between the variables may affect the truth of the regression, this study carried out the correlation analysis of each variable to exclude the strong correlation between the variables. The test results are shown in Table 3. It is generally believed that a strong correlation exists when the correlation coefficient between variables is greater than or equal to 0.8.

**Table 3:** Correlation analysis test results.

	patent	sub	tax	size	rd	cura	lev	GDP
patent	1							
sub	0.856***	1						
tax	0.832***	0.316***	1					
size	0.385**	0.124**	0.316**	1				
rd	0.238**	−0.017	0.444**	0.374**	1			
cura	−0.0812**	−0.051**	−0.172**	−0.425**	−0.185**	1		
lev	0.147**	0.064**	0.223**	0.543**	0.143**	−0.746**	1	
GDP	0.016	−0.193**	0.191**	−0.112**	0.09*	0.045*	−0.062**	1

***, **, and * denote significance at the 1, 5, and 10% significance levels, respectively.

The results show that the correlation coefficients of government subsidies, tax incentives, enterprise size, R&D investment, gearing ratio, and the economic environment in which the enterprise is located with green technological innovation and green enterprise development are positive, while the correlation coefficient of current ratio with green technological innovation and green enterprise development is negative. Specifically, the correlation coefficient of sub with green technology innovation and green enterprise development is 0.856, which indicates that the role of government subsidies on green technology innovation and green enterprise development is positively promoted and is significant at 1% level, indicating that the government subsidies have a significant positive correlation with green technology innovation and green enterprise development. The correlation coefficient of TAX with green technology innovation and green enterprise development is 0.832 and is significant at a 1% level of significance. This indicates that the impact of tax incentives on green technology innovation and green enterprise development is also positively promoted, and the promotion effect is very significant. Observing the control variables shows that the correlation coefficients of enterprise size, R&D investment, and gearing ratio with green technological innovation and green enterprise development are 0.385, 0.238, and 0.147, respectively, and are significant at the 5% level. It indicates that these control variables have a relatively significant positive impact on green technology innovation and green enterprise development. The correlation coefficient between the GDP of the region where the enterprise is located and the quality of innovation is positive, but it does not show a more significant relationship, and subsequent regression verification is needed. The current ratio is significantly negatively correlated with green technological innovation and green enterprise development and plays an inhibitory role. This is most likely due to the backlog of inventory, accounts receivable, long collection time, and the increase in pending property losses. The enterprise can be used to repay debt, but cash deposit is seriously short, inhibiting the enterprise’s innovative activities.

The study is further analyzed using the multicollinearity test, the results of which are reported in Table 4. VIF stands for Variance Inflation Factor for all variables. It is generally accepted that the variance inflation factor is less than 10 to consider the absence of multicollinearity. Table 4 shows that the variance inflation factors of all variables are less than or equal to 2.685, and the overall variance inflation factor is only 1.710, so it can be determined that the model does not have multicollinearity.

**Table 4:** Multicollinearity test.

Variables	VIF	1/VIF
sub	1.161	0.964
tax	1.352	0.817
size	1.826	0.594
rd	1.364	0.809
cura	2.365	0.455
lev	2.685	0.399
GDP	1.214	0.918
Mean VIF	1.710	

### 4.3 Benchmark regression analysis

According to the econometric model of Equations 1–3, econometric estimation of the sample data is carried out to obtain the regression results of the impact of government subsidies and tax incentives on the technological innovation and development of listed enterprises in China’s new energy industry, as shown in Table 5.

**Table 5:** Impact of fiscal and tax incentive policies on technological innovation and development of listed enterprises in the new energy industry.

Variables	(1)	(2)	(3)
	patent	patent	patent
sub	0.0085***		0.0083**
	(2.31)		(2.33)
tax		0.0152***	0.0146**
		(1.99)	(1.92)
size	0.2738**	0.2585**	0.2589**
	(5.68)	(5.35)	(5.33)
rd	0.1114**	0.0996**	0.0993**
	(4.69)	(4.75)	(4.66)
cura	−0.0253	−0.0198	−0.0245
	(−0.74)	(−0.61)	(−0.72)
lev	−0.5180*	−0.5353*	−0.5167*
	(−1.93)	(−1.95)	(−1.90)
GDP	0.2961**	0.2142**	0.2612**
	(4.31)	(3.23)	(3.75)
Constant	−15.3388***	−12.7664***	−14.1462***
	(−6.95)	(−5.77)	(−6.18)
Wald chi2	142.65***	140.23***	144.05***

***, **, and * denote signifcance at the 1, 5, and 10% signifcance levels, respectively.

In Table 5, column (1) is the result of regression analysis of model (1). The table shows that the regression coefficient of government subsidy (sub) is 0.0085, indicating that government subsidy is positively related to green technology innovation and green enterprise development. It is significant at a 1% significance level, which indicates that the government subsidy policy can effectively enhance the technological innovation and development of listed enterprises in the new energy industry, and hypothesis H1 is valid. Column (2) is the result of the regression analysis of the model (2). The table shows that the regression coefficient of tax preference (TAX) is 0.0152, indicating that the tax preference policy is positively correlated with green technology innovation and green enterprise development. The regression coefficient is 0.0152, which indicates that tax incentives are positively related to green technological innovation and green enterprise development. It is significant at the 1% level, which indicates that tax incentives can effectively enhance the technological innovation and development of listed enterprises in the new energy industry, and hypothesis H2 is established. Column (3) is the result of regression analysis of model (3). As can be seen from the table, the regression coefficients of the two explanatory variables, government subsidies (sub) and tax incentives (tax), are both positive and significant at the 5% level, and hypothesis H3 holds. Based on the above analysis, it can be seen that the regression results of this study are consistent with hypotheses H1, H2, and H3.

### 4.4 Robustness test

To ensure the reliability of the above regression analysis results, this study first adopts the method of reducing control variables for the robustness test. The regression analysis is performed again by eliminating the control variable cura, resulting in the robustness test results in Table 6. Observing the data in the table, we can observe that the regression coefficients of the explanatory variable government subsidies in the model (1) and the explanatory variable tax incentives in model (2) are both positive, and both are significantly positive at the 1% level. This indicates that both government subsidies and tax incentives can significantly promote technological innovation and the development of listed enterprises in the new energy industry. The regression coefficients of the two explanatory variables, government subsidies (sub) and tax incentives (tax), in Column (3) are both positive and significant at the 5% level, indicating that both government subsidies and tax incentives can jointly promote technological innovation and development of listed enterprises in the new energy industry. The robustness test results in Table 6 are consistent with the previous conclusions, and thus, the conclusions are robust.

**Table 6:** Robustness test results of model (1–3).

Variables	(1)	(2)	(3)
	patent	patent	patent
sub	0.0089***		0.0085**
	(2.32)		(2.31)
tax		0.0155***	0.0149**
		(2.03)	(1.95)
size	0.2428**	0.2285**	0.2183**
	(5.34)	(4.89)	(4.87)
rd	0.1052**	0.1031**	0.1037**
	(4.85)	(4.82)	(4.81)
lev	0.0253	0.0196	0.0148
	−0.55	−0.77	−0.65
GDP	0.2961**	0.2142**	0.2612**
	(4.31)	(3.12)	(3.66)
Constant	−14.8688***	−12.2396***	−13.6675***
	(−6.76)	(−5.57)	(−5.94)
Wald chi2	137.69***	135.53***	

***, **, and * denote signifcance at the 1, 5, and 10% signifcance levels, respectively.

### 4.5 Heterogeneity analysis

This study divides the sample enterprises into East, Central, and West regions to examine the difference in the impact of fiscal and tax incentive policies on the technological innovation and development of listed enterprises in the new energy industry located in different levels of economic zones and yields regression results as shown in Table 7.

**Table 7:** Regression results of regional heterogeneity.

Variables	East	Central	West
	patent	patent	patent
sub	0.0092**	0.132***	0.0157***
	(0.76)	(1.99)	(2.11)
tax	0.0145**	0.0156***	0.0425***
	(1.86)	(2.23)	(3.16)
size	0.2655**	0.2779**	0.2018**
	(4.71)	(2.67)	(1.92)
rd	0.0816**	0.0845**	0.1486**
	(3.56)	(3.62)	(3.07)
cura	−0.0336	−0.0572	−0.0463
	(−0.92)	(−0.67)	(−0.58)
lev	0.0096	0.0137	0.0235
	(0.74)	(0.58)	(0.49)
GDP	0.2834	0.5205	0.7028**
	(1.85)	(3.35)	(4.63)
Constant	−8.5662***	−21.4287***	−27.5315***
	(−2.69)	(−4.62)	(−6.13)
Wald chi2	74.68***	79.84***	83.65***
Observations	1,276	575	337

***, **, and * denote signifcance at the 1, 5, and 10% signifcance levels, respectively.

As can be seen from the data in Table 7, Column (1) is the sample regression result of the eastern provinces. The regression coefficient of government subsidy (sub) in the regression result of Column (1) is 0.0092, and the regression coefficient of tax incentives (tax) is 0.0145. Both of them are significantly positive at the 5% level. It indicates that in the eastern region, government subsidies and tax incentives facilitate the technological innovation and development of listed enterprises in the new energy industry. Columns (2) and (3) show the sample regression results for central and western provinces. The regression coefficient of government subsidies (sub) in column (2) is 0.0132, which is significantly positive at the 1% level. The regression coefficient of tax incentives (tax) in column (3) is 0.0425, which is also significant at the 1% level, indicating that tax incentives in the central and western regions can significantly enhance the technological innovation and development of listed enterprises in the new energy industry. The reason for this may lie in the economic development of the eastern region being better, and the listed enterprises in the new energy industry in this region are also in good business conditions. They face less financial pressure on innovation and R&D activities and have enough funds to invest in green technology innovation. The economic development of the central and western regions is relatively backward. The green, low-carbon industry in the region may be relatively difficult to finance, which will impede the enterprise’s green technology innovation and development. Government subsidies and tax incentives give listed enterprises in the new energy industry an opportunity for further development. Implementing government subsidies and tax incentives will substantially increase enterprises’ available capital, improve enterprises’ enthusiasm in innovation and research and development, and then green technology innovation and development. Therefore, government subsidies and tax incentives have a greater impact on listed enterprises in the central and western regions of the new energy industry.

### 4.6 Comparative analysis with alternative models

To evaluate the sensitivity of the green innovation output of the proposed model to fiscal and tax incentives, it is compared with two other alternative models (The fixed effects model) and the panel model with interactions. The results are shown in Table 8.

**Table 8:** Comparative results of model performance and policy elasticity.

Model	Adjusted R^2^	Coefficient of sub (gov. subsidy)	Coefficient of tax (tax incentive)	Policy elasticity (%)
Proposed model	0.82	0.0085 (*p* < 0.01)	0.0152 (*p* < 0.01)	12.8
Fixed effects model	0.75	0.0069 (*p* < 0.05)	0.0107 (*p* < 0.05)	8.5
Panel model with interactions	0.78	0.0074 (p < 0.01)	0.0135 (*p* < 0.01)	10.2

The *p*-values in parentheses indicate the statistical significance of the coefficients. A *p*-value of < 0.01 signifies a highly significant relationship at the 1% level, while a *p*-value of < 0.05 indicates statistical significance at the 5% level.

As can be seen from Table 8, the regression model in this study explains 82% of the variance of green technology innovation, which is better than the other two comparison models. At the same time, in terms of fiscal subsidies (sub) and tax incentives (tax), the proposed model shows stronger and more significant positive effects than the alternatives. The elasticity measure results show that the model identifies fiscal and tax incentives as the more influential drivers of green innovation.

## 5 Development mechanism of green environmental technology and green enterprises from the perspective of government fiscal and tax incentives

### 5.1 Fully exploit government fiscal and tax incentive policies to maximize enterprise R&D funds

Generally, the government tends to lag behind market demand when making tax incentive policies. Therefore, green enterprises are involved in researching and developing green environmental protection technologies, especially those addressing the emergence of new high-tech waste. It is difficult to obtain substantial and corresponding fiscal and tax preferences from the government at the project’s establishment. Therefore, green enterprises should combine their own conditions to fully explore various fiscal and tax incentive policies issued and implemented by government departments, such as tariff exemption, pre-tax deduction of wages, rapid depreciation of equipment, etc., to maximize the enjoyment of the government’s fiscal and tax preferential policies. At the same time, due to the high risk and long cycle of green technology research and development, most enterprises will retain core technology information in the research and development process. It is difficult for government departments and financial institutions to understand the specific content of green technology research and development in detail, making it difficult for enterprises to obtain support from external funds. In this context, enterprises can collaborate with government departments or apply for various funding projects offered by the government to obtain policy support. They can also consider extending some projects that can be postponed appropriately and strive for targeted fiscal and tax incentive policies implemented by the government departments during this period. Additionally, enterprises should take the initiative to monitor the R&D funds allocated for green and environmental protection technologies. For government subsidy funds, enterprises should strictly check and use them carefully to improve their reputation in government departments and obtain more financial support.

While government fiscal and tax incentive policies are crucial for supporting green enterprises, their implementation presents several potential challenges. These include delayed policy adaptation to rapidly changing market needs, difficulties in securing external financing due to the high risks and uncertainties of green technology R&D, and complex administrative requirements that hinder access to incentives. Additionally, the risk of technology spillover discourages investment in innovation, while policymakers often lack a comprehensive understanding of green technologies, resulting in inadequate support. Inconsistent fiscal and tax incentives across regions further create an uneven playing field. To address these challenges, green enterprises should engage in ongoing dialogue with policymakers to advocate for timely updates to incentives and develop flexible R&D timelines. They can enhance transparency in R&D to secure financing, invest in specialized teams to navigate administrative complexities, and prioritize intellectual property protection to mitigate technology spillover risks. Active participation in policy discussions can help shape more effective incentives, and expanding operations into regions with favorable policies while diversifying funding sources can provide more consistent support.

### 5.2 Formulate long-term development plans for green technology and enterprises based on public interests

**Figure 3:**
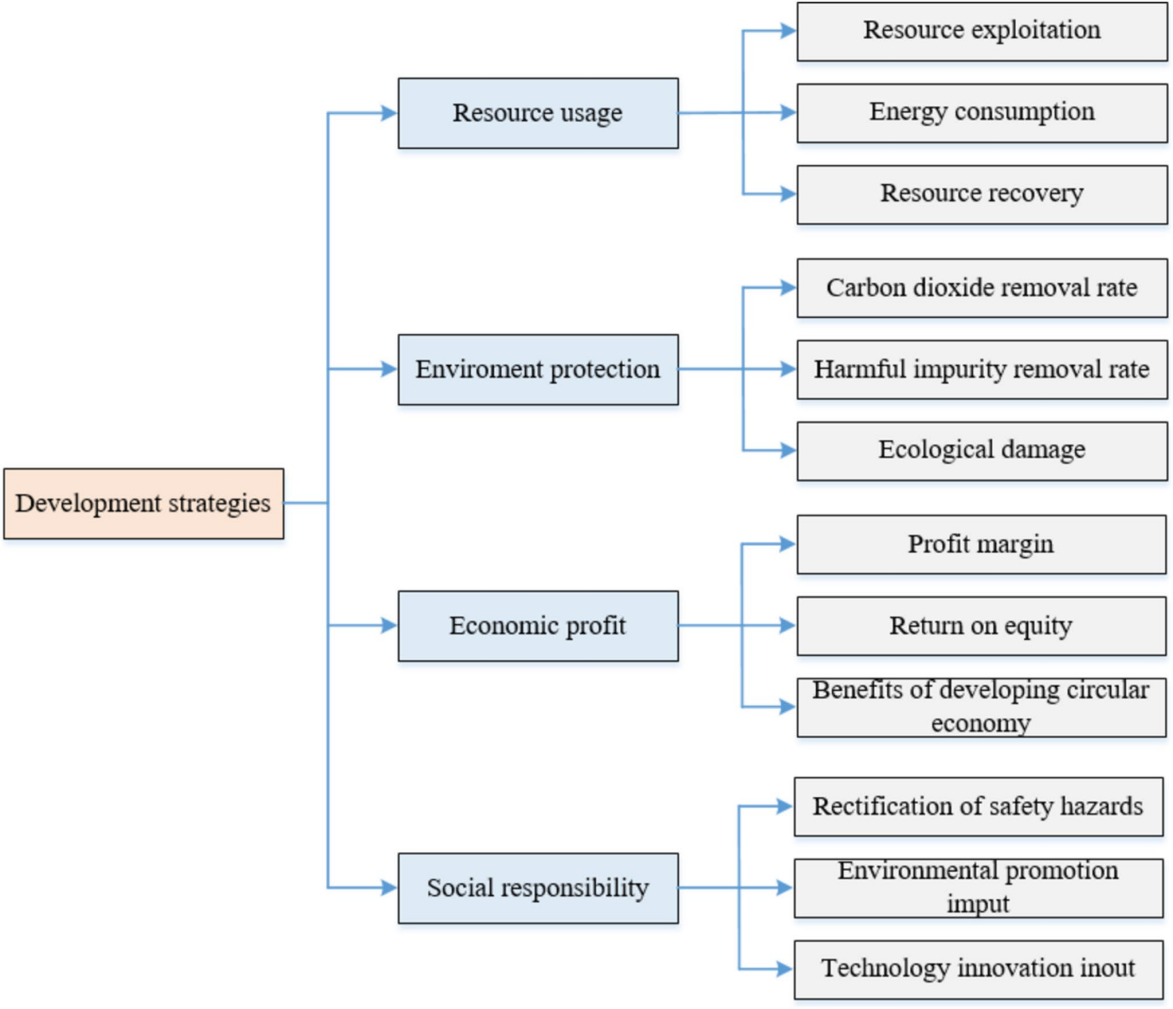
Essence of the developing strategies for a green enterprise.

Research, development, and application of green technology innovation fall into the category of public welfare, often yielding greater societal benefits than profits. Therefore, when creating development plans, green enterprises should consider long-term perspectives and public interests, aligning their strategies with the broader framework of low-carbon economic development in their country or region. This approach, as shown in Figure 3, makes it easier to obtain government support. For example, the emergence of new industrial waste can lead to public health issues. If a green enterprise prioritizes public interest and commits to researching the handling and disposal of industrial waste, it will gain widespread government and public support. Once developed, such innovations can have significant market potential and benefits. At the same time, when making development plans, enterprises should put the public interest first to find opportunities for green technology research and innovation. From a public health perspective, rapid industrialization in recent years has introduced many new materials that are difficult to degrade, leading to environmental pollution. Some of these materials, once abandoned, can accumulate in the bodies of animals and plants as tiny particles. When these organisms are processed into food, these particles can accumulate in the human body and not be digested, resulting in health problems. Therefore, if the enterprise can devote itself to research on the degradation treatment or recycling of new materials, it can effectively solve the public health problems people face. At the same time, it can also obtain strong support from the government and obtain the corresponding government fiscal and tax preferential policies.

To further integrate public health and sustainable development into their long-term strategies, enterprises should consider the following guidelines:

1. Assess environmental and health impact: Enterprises should identify how their activities impact the environment and public health, using tools like lifecycle analysis to quantify pollution and health risks, allowing for targeted improvements.
2. Align innovation with health solutions: Green technology development should address public health issues, such as investing in waste treatment or recycling technologies that reduce pollutants, benefiting society and the enterprise’s market position.
3. Set measurable sustainability goals: Enterprises should set clear goals, like reducing emissions and optimizing energy use, while tracking health-related outcomes to ensure alignment with public welfare.
4. Collaborate with authorities: Partnering with government and health agencies helps enterprises align with national environmental goals and gain policy and technical support.
5. Integrate CSR with health goals: CSR initiatives should include public health efforts, such as community education and green infrastructure, to enhance both social image and local health outcomes.
6. Leverage demand for sustainable products: Green enterprises should capitalize on the growing market for sustainable products by educating consumers, improving product acceptance, and fostering business growth alongside public benefits.

### 5.3 Attach importance to the innovation and promotion of green technology and increase the publicity and education of green development

Green technology is a new technology conducive to resource conservation, environmental governance, and ecological protection. It has considerable frontier and complexity. However, once the research and development is successful, it can occupy the market and obtain considerable benefits. As a result, the world will fully use its regions and technological advantages and choose different areas to obtain key technological breakthroughs. For example, Sweden chooses mainly to develop environmental technology, and its environmental technology is at the world-advanced level, not only in the service of the country’s environment but also vigorously promotes the green development of other countries through environmental technology exports. Denmark focuses on the development of energy technology, which is at the forefront of the world and provides technical support for the development of a new energy industry. At the same time, the export of energy technology is increasing year by year, making the energy industry the main economic growth point of Denmark. With the advancement of industrialization and technological processes, there will be more and more industrial products, and how to deal with the waste of these industrial products after they are abandoned will be one of the important directions of future research on green technology innovation. If a green enterprise can combine its own advantages for technology innovation research while seeking government support, it will form its own technological advantages in future industrial development and drive regional economic growth.

In addition, the cost of green environmental protection technology research and development is higher, so the cost of green product marketing is also higher for the market and consumers, and in a short period of time, it may be difficult to accept. This resistance can be attributed to several factors: (1) Consumer price sensitivity: Many consumers prioritize cost when purchasing. Green products typically involve more expensive materials and production processes and are often priced higher than their conventional counterparts. This price differential can deter price-sensitive consumers, especially in markets with limited disposable income. As a result, even if the long-term benefits of green products, such as energy savings and health advantages, are communicated effectively, the initial higher costs can lead to reluctance to adopt. (2) Perceived value: The perceived value of green products plays a crucial role in consumer acceptance. If consumers do not fully understand these products’ environmental and health benefits, they may be less inclined to pay the premium. Effective marketing strategies that emphasize the long-term benefits and sustainability of green products are essential in reshaping consumer perceptions and justifying the higher costs. (3) Lack of awareness and education: A significant barrier to the acceptance of green products is the general lack of awareness and education surrounding environmental issues. Many consumers may not recognize the importance of reducing their carbon footprint or the impact of their purchasing decisions on the environment. Initiatives such as public awareness campaigns, educational programs in schools, and community engagement activities can help bridge this knowledge gap, fostering a greater understanding of the benefits of green products and encouraging more sustainable consumption behaviors.

Therefore, the government and enterprises should attach importance to green development through publicity and promotion, such as extensive campus green education and outdoor green education practice, cultivate the consciousness and behavior of green development, and then create a good social atmosphere for green development for enterprises to carry out green innovation and development to lay a good social foundation. For example, enterprise propaganda activities can be held on state or local public health days, letting citizens realize the emergence of new industrial waste and its harm to public health and urging citizens to reduce environmental pollution. Enterprises can also invite students and teachers to visit the production line, promoting green development concepts and letting more students join in the green environmental protection technology research and development in my career.

## 6 Conclusion

This study addresses contemporary challenges by exploring how government fiscal and tax incentives can promote the development and application of green technologies, ultimately fostering corporate environmental responsibility and improving public health and sanitation. Using empirical data from listed enterprises in the new energy automotive industry from 2018 to 2023, the study uses a multiple regression model to assess the impact of government subsidies and tax incentives on green technology innovation and firm growth while controlling for factors such as firm size and R&D investment. Compared with other methodologies, such as fixed effects and panel interaction models, the results show that our approach demonstrates superior explanatory power (Adjusted R^2^ = 0.82) and reveals stronger positive impacts of government subsidies and tax incentives on green innovation (elasticity: 12.8%). This comparison underscores the strength of the proposed model in capturing the nuanced effects of fiscal policies on green enterprises. By offering recommendations such as flexible tax incentives, industry-specific subsidies, and improved collaboration platforms, this study provides a roadmap for overcoming these challenges and enhancing the effectiveness of green policies. In terms of fulfilling the research objective, this study offers a comprehensive understanding of the ways in which fiscal and tax incentives can accelerate the adoption of green technologies. Additionally, by considering regional disparities in policy effectiveness and proposing targeted solutions, the study attempts to address the varying needs of enterprises based on their size and geographic location, contributing to more equitable and effective policy outcomes.

To make the findings more broadly impactful, future research will aim to expand the scope of the study by exploring how our findings relate to the experiences of other countries, particularly those that have been successful in environmental and energy technologies. Additionally, a more detailed analysis will be conducted to examine the different obstacles enterprises of varying sizes face when accessing preferential policies. Furthermore, the limitations of this study, such as its focus on a single industry and regional context, suggest that future studies should consider the broader application of fiscal and tax incentives across various sectors and regions. Investigating the specific challenges faced by small, medium, and large enterprises in utilizing these incentives will provide valuable insights into the effectiveness of these policies in promoting green technology innovation on a global scale.

## Data Availability

The original contributions presented in the study are included in the article/supplementary material. Further inquiries can be directed to the corresponding author.
